# Crystal Crosslinked Gels for the Deposition of Inorganic Salts with Polyhedral Shapes

**DOI:** 10.3390/gels4010016

**Published:** 2018-02-06

**Authors:** Yumi Mochizuki, Chihiro Oka, Takumi Ishiwata, Kenta Kokado, Kazuki Sada

**Affiliations:** 1 Graduate School of Chemical Sciences and Engineering, Hokkaido University, Sapporo, Hokkaido 060-0810, Japan; sadalab01mdpi@gmail.com (Y.M.); sadalab02mdpi@gmail.com (C.O.); sadalab03mdpi@gmail.com (T.I.); 2 Faculty of Science, Hokkaido University, Sapporo, Hokkaido 060-0810, Japan

**Keywords:** biomineralization, calcium carbonate, calcium phosphate, metal-organic framework, crystal crosslinking

## Abstract

Biomineralization has been given a great deal of attention by materials chemists because of its low environmental load and sustainability. With the goal of synthesizing such processes, various methods have been developed, especially for inorganic salts of calcium. In this report, we focused on the deposition of inorganic salts, such as calcium carbonate and calcium phosphate using crystal crosslinked gels (CCG), which are prepared by crystal crosslinking of metal-organic frameworks (MOFs). Due to the crystalline nature of MOFs, CCGs intrinsically possess polyhedral shapes derived from the original MOF crystals. As the result of deposition, the obtained inorganic salts also exhibited a polyhedral shape derived from the CCG. The deposition mainly occurred near the surface of the CCG, and the amorphous nature of the deposited inorganic salts was also confirmed.

## 1. Introduction

Organisms have developed various biominerals by hierarchically combining soft and hard materials with elaborate structural designs in multiscale, to achieve greater performance than the single components [1,2]. For the inorganic salt for the scaffold, they usually employ calcium carbonate, hydroxyapatite, silicate, and iron oxide. For example, some mollusks produce nacre as an inner shell layer from calcium carbonate and biopolymers including a chitin layer and proteins, which is strong, resilient, and iridescent [3].

From the viewpoint of sustainability and low environmental loads, such biomineralization processes have attracted great attention from material scientists, and various systems to control the crystallization process of inorganic salts have been explored up to the present, when synthetic polymers were added in the crystallization condition [4,5,6,7,8,9,10,11]. For instance, Kato and coworkers reported the preparation of calcium carbonate thin film by utilizing an insoluble polymer matrix as the substrate and soluble acidic polymer as the additive [12,13,14,15]. Chujo and coworkers studied the control of crystal structure and particle size of calcium carbonate by employing poly(amidoamine) (PAMAM) dendrimer as the additive in the crystallization condition [16,17,18]. Estroff and coworkers demonstrated crystal growth control of calcium carbonate by using agarose gel and consequent production of porous crystal of calcite after calcination, due to effective incorporation of gel matrix in the crystal [19,20,21,22]. These studies revealed that the molecular structure of the additive in the crystallization condition plays a crucial role for the crystallization process of the inorganic salts including calcium carbonate. They also suggested possible ways to control the shape of obtained inorganic salts [23], which would lead to other control methods for the crystallization process of such inorganic salts. 

Recently, we have developed a new methodology to obtain polymer gels with well-defined structures derived from template crystals, the crystal crosslinking method [24,25,26,27,28,29]. The method involves the post synthetic modification (PSM) of metal-organic frameworks (MOF); the resulting crystal crosslinking gels (CCGs) possessed a polyhedral shape derived from the original MOF crystals. Additionally, after the hydrolysis of crosslinked MOFs for removal of metal cations, abundant carboxylate groups remained inside the CCGs, which was shown using the absorption test for metal cations [29]. The abundant carboxylate groups in a CCG can attract other metal cations such as Ca^2+^, thus the later deposition of inorganic salts will occur around the carboxylate groups in the CCG. In this study, we demonstrated the deposition of inorganic salts such as calcium carbonate and calcium phosphate by using CCG as the shape-directing agent. The deposited inorganic salts should possess a well-defined shape derived from the employed CCG, and therefore from the original MOF crystal.

## 2. Results and Discussion

### 2.1. Preparation 

**Figure 1:**
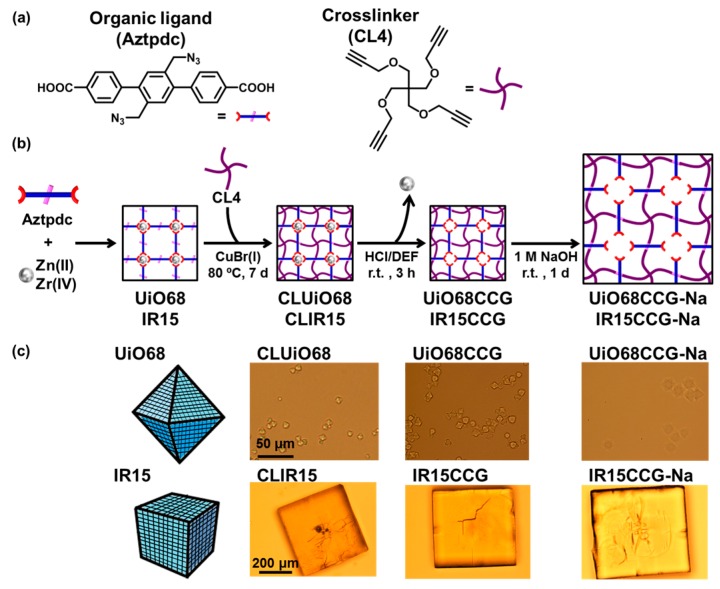
(**a**) Molecular structures of the reactive organic ligand Aztpdc and crosslinker CL4; (**b**) Schematic image of preparation route for CCG-Nas (UiOCCG-Na and IR15CCG-Na); (**c**) Appearance of crosslinked MOFs (CLUiO68 and CLIR15), CCGs (UiO68CCG and IR15CCG), and CCG-Nas (UiOCCG-Na and IR15CCG-Na).

As the shape-directing agent, we selected crystal crosslinked gels (CCGs) derived from UiO-68 or IRMOF15 (IR15). Therefore, AzUiO68 and AzIR15 were first obtained from Aztpdc as the reactive organic ligand and metal ions, Zr(IV) and Zn(II), respectively (Figure 1a,b). Then the Aztpdc in the crystals were crosslinked with a multialkynyl crosslinker CL4 to provide CLUiO68 and CLIR15, which were transformed to corresponding CCGs, UiO68CCG and IR15CCG, via acidification. The carboxylic acid groups inside the CCGs were then neutralized under basic conditions to obtain a sodium carboxylate analogue (CCG-Nas). The CCG-Nas showed greater swelling compared to that of CCGs (Figure 1c), due to the electrostatic repulsion between the carboxylate groups and osmotic pressure derived from their polyelectrolyte gel property. The ion exchange capacity of IR15CCG-Na was determined as 2.28 meq/g for Ca^2+^ (76 mol% exchange) by inductively coupled plasma-atomic emission spectroscopy (ICP-AES). The inorganic calcium salt, calcium phosphate or calcium carbonate, was deposited on CCG-Nas by immersion in Tris-HCl buffer (PH = 7.4) containing 200 mM CaCl_2_ at first, and then immersion in 120 mM Na_2_HPO_4_ aq. or 200 mM Na_2_CO_3_ aq. This cycle was repeated three times to thicken the deposited calcium salt (see Figure S1 in Supplementary Materials). For the case of UiO68CCG-Na, the dispersed particle was collected by centrifuge, whereas the sample from UiO68CCG-Na was collected by filtration.

### 2.2. Calcium Phosphate Deposited on CCG-Nas 

**Figure 2:**
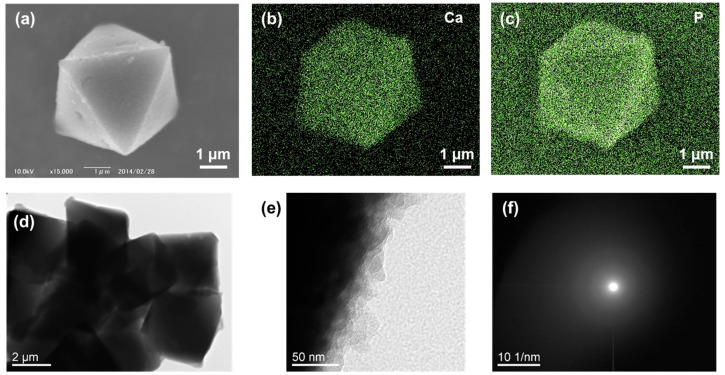
(**a**) SEM image of calcium phosphate deposited on UiO68CCG-Na (UiO68CCG-Na@CP); EDX mapping of (**b**) Ca and (**c**) P atoms for image (**a**); (**d**) TEM image of UiO68CCG-Na@CP; (**e**) the magnified image; (**f**) SAED observation from image (**e**).

Scanning electron microscopy (SEM) revealed the well-defined regular octahedron shape of UiO68CCG-Na@CP with ~5 μm side length after deposition of calcium phosphate (Figure 2a). Energy dispersive X-ray spectrometry (EDX) analysis showed the presence of Ca and P atoms in the whole area of the particle of UiO68CCG-Na@CP (Figure 2b,c). The deposited calcium phosphate mainly existed at the vicinity of particle’s surface, shown by line analysis of EDX (Figure S2a). The abundant carboxylate groups inside CCG-Na effectively prevented the penetration of phosphate anion to the core of CCG-Na, while cationic Ca^2+^ would be distributed in the whole range of the particle (Figure S2). Transmission electron microscopy (TEM) also confirmed the regular octahedron shape of the particle and the deposition of calcium phosphate on the surface (Figure 2d,e). No distinct patterns were observed in selected area electron diffraction (SAED) analyses, indicating the amorphous nature of the deposited calcium phosphate (Figure 2f).

**Figure 3:**
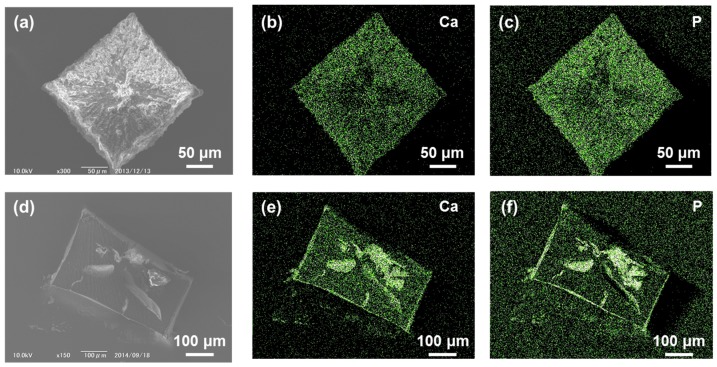
(**a**) SEM image of calcium phosphate deposited on IR15CCG-Na (IR16CCG-Na@CP); EDX mapping of (**b**) Ca and (**c**) P atoms for image (**a**); (**d**) SEM observation of cross-section of IR16CCG-Na@CP; EDX mapping of (**e**) Ca and (**f**) P atoms for image (**d**).

As with the case of UiO68CCG-Na, the deposition of calcium phosphate on IR15CCG-Na (IR15CCG-Na@CP) was achieved while keeping the cubic shape of the original CCG-Na, as shown in Figure 1a. EDX analysis showed the full distribution of Ca and P atoms in the whole area of the particle of IR15CCG-Na@CP (Figure 3b,c). The deposition of calcium phosphate occurred mainly in the vicinity of surface and cracks, indicated by the EDX analyses of cross-sections (Figure 3d–f). Powder X-ray diffraction showed no obvious patterns (confirming the amorphous nature of the deposited calcium phosphate).

### 2.3. Calcium Carbonate Deposited on CCG-Nas 

**Figure 4:**
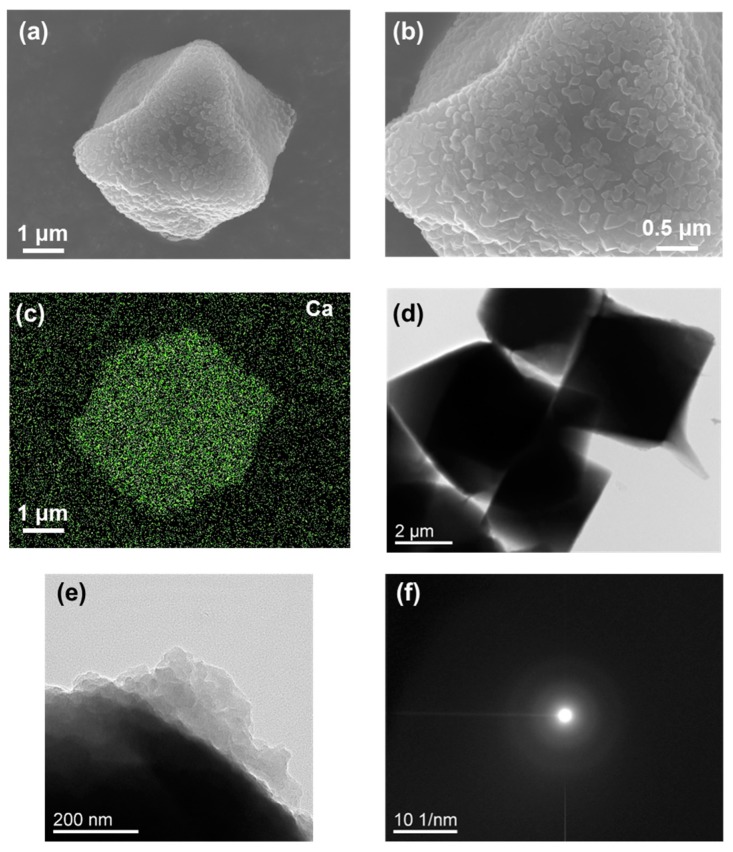
(**a**) SEM image of calcium carbonate deposited on UiO68CCG-Na (UiO68CCG-Na@CC); (**b**) Magnified SEM image from image (**a**); (**c**) EDX mapping of Ca atoms for image (**a**); (**d**) TEM image of UiO68CCG-Na@CC; (**e**) the magnified image; and (**f**) SAED observation from image (**e**).

The deposition of calcium carbonate on UiO68CCG-Na was confirmed by SEM observation as shown in Figure 4a,b. After the deposition, the regular octahedron shape of the original CCG-Na was retained. The full distribution of Ca atoms was checked by EDX analysis (Figure 4c), although the distribution of carbonate cannot be shown due to its similar atomic composition to the original CCG-Na. Thus, the line analysis of EDX revealed only the full distribution of Ca atoms in the whole area of the particle (Figure S2b). On the basis of the analogy with calcium phosphate, the deposition should mainly occur in the vicinity of the particle due to the electrostatic repulsion between carbonate anions and abundant carboxylate groups inside CCG-Na. TEM observation also demonstrated the regular octahedron shape of the particle and the deposition of calcium carbonate on the surface (Figure 4d,e). SAED analysis showed no distinct patterns, indicating the amorphous nature of the deposited calcium carbonate (Figure 4f).

**Figure 5:**
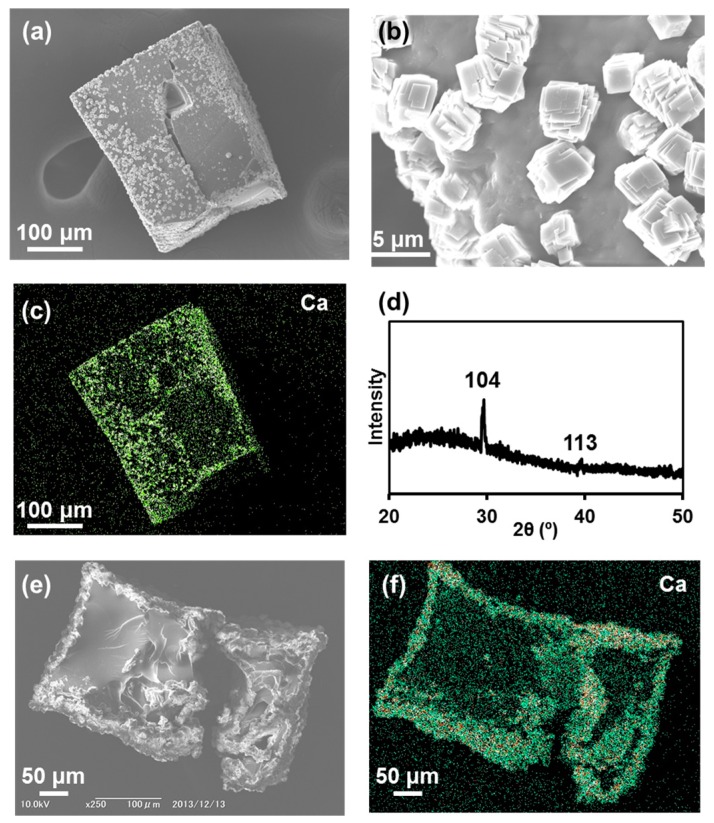
(**a**) SEM image of calcium carbonate deposited on IR15CCG-Na (IR15CCG-Na@CC); (**b**) Magnified SEM image from image (**a**); (**c**) EDX mapping of Ca atom for image (**a**). (**d**) XRD pattern of IR15CCG-Na@CC; (**e**) SEM observation of cross-section of IR15CCG-Na@CC. EDX mapping of (**f**) Ca atom for image (**e**).

The deposition of calcium carbonate on IR15CCG-Na was achieved while keeping the cubic shape of the original CCG-Na, as with the case of UiO68CCG-Na. From the SEM observation, the production of small cubic particles with ~3 μm side length on the surface of CCG-Na was confirmed (Figure 5a,b). EDX analysis showed the presence of Ca atoms in the small cubic particle (Figure 5c). X-ray diffraction (XRD) measurement exhibited distinct patterns at 29.6° and 39.6°, which are typical for (104) and (113) of calcite (Figure 5d). Additionally, Fourier transform infrared spectroscopy (FT-IR) showed the characteristic peak of calcite at 875 cm^−1^ (Figure S3). SEM observation and EDX analysis of the cross-section of IR15CCG-Na@CC indicated the predominant deposition of calcium carbonate in the vicinity of surface and cracks (Figure 5e,f). The rapid crystallization kinetics of calcium carbonate would lead to the observed calcite formation on CCG-Na.

## 3. Conclusions

In this study, we succeeded in the hybridization of inorganic salts such as calcium carbonate and calcium phosphate with polyhedral crystal crosslinked gels (CCGs) via simple alternate immersion method of CCGs in aqueous solution of calcium cations and the corresponding anions. The polyhedral shape of CCG was preserved even after the deposition of the inorganic salt, and the deposition occurred predominantly in the vicinity of the surface of the CCG, as shown by EDX line analysis. The deposited inorganic salts were found to be amorphous, rather than the combination of calcium carbonate and IR15CCG-Na. Our approach is a promising way to control the shape of inorganic salts.

## 4. Materials and Methods

### 4.1. General

UiO68CCG and IR15CCG were synthesized via a previously published process [28]. Other reagents and solvents were purchased from commercial sources and used without further purification. All experiments were carried out in the ambient atmosphere, unless otherwise mentioned. Powder X-ray diffraction (XRD) patterns were obtained using a Bruker AXS D8 ADVANCE (Bruker Corporation, Billerica, MA, USA). Field emission scanning electron microscope (FE-SEM) observations and energy dispersive X-ray spectrometry (EDX) analyses were carried out via a JEOL JSM-6500F (JEOL Ltd., Akishima, Japan). Transmission electron microscope (TEM) observations and selected area electron diffraction (SAED) analyses were carried out on a JEOL JEM-2010 (JEOL Ltd., Akishima, Japan). Fourier transform infrared (FT-IR) spectra were recorded on a JASCO FT/IR-4100 spectrometer (JASCO Corporation, Hachioji, Japan). Inductively coupled plasma atomic emission spectroscopy (ICP-AES) was carried out by a Shimadzu ICPE-9000 instrument (Shimadzu Corporation, Kyoto, Japan).

### 4.2. Preparation of UiO68CCG-Na

UiO68CCG (25 mg) was immersed in 1 M NaOH aq. (2 mL) for 3 h at room temperature. Then it was washed with distilled water (three times) and diethylformamide (DEF, three times), using a centrifuge (10,000 rpm, 5 min).

### 4.3. Preparation of IR15CCG-Na

IR15CCG (25 mg) was immersed in 1 M NaOH aq. (2 mL) for 3 h at room temperature. Then it was washed with distilled water (three times) and diethylformamide (DEF, three times) via decantation.

### 4.4. Preparation of UiO68CCG-Na@CP

UiO68CCG-Na (20 mg) was firstly immersed in Tris-HCl buffer (PH = 7.4, 2 mM) containing 200 mM CaCl_2_ for 5 min, then it was centrifuged (10,000 rpm, 5 min), and washed with distilled water for 5 min followed by another centrifuging (10,000 rpm, 5 min). Then it was immersed in 120 mM Na_2_HPO_4_ aq. for 5 min, centrifuged (10,000 rpm, 5 min), and washed with distilled water for 5 min followed by another centrifuging (10,000 rpm, 5 min). This cycle was repeated three times.

### 4.5. Preparation of IR15CCG-Na@CP

IR15CCG-Na (20 mg) was first immersed in Tris-HCl buffer (PH = 7.4, 2 mM) containing 200 mM CaCl_2_ for 5 min, followed by decantation, and washed with distilled water for 5 min followed by decantation. Then it was immersed in 120 mM Na_2_HPO_4_ aq. for 5 min, decanted, and washed with distilled water for 5 min, followed by decantation. This cycle was repeated three times.

### 4.6. Preparation of UiO68CCG-Na@CC

UiO68CCG-Na (20 mg) was first immersed in Tris-HCl buffer (PH = 7.4, 2 mM) containing 200 mM CaCl_2_ for 5 min, then it was centrifuged (10,000 rpm, 5 min), and washed with distilled water for 5 min followed by centrifuging (10,000 rpm, 5 min). Next, it was immersed in 200 mM Na_2_CO_3_ aq. for 5 min, then centrifuged (10,000 rpm, 5 min), and washed with distilled water for 5 min followed by centrifuging (10,000 rpm, 5 min). This cycle was repeated three times.

### 4.7. Preparation of IR15CCG-Na@CC

IR15CCG-Na (20 mg) was first immersed in Tris-HCl buffer (PH = 7.4, 2 mM) containing 200 mM CaCl_2_ for 5 min followed by decantation, then washed with distilled water for 5 min followed by decantation. Then it was immersed in 200 mM Na_2_CO_3_ aq. for 5 min, then decanted, washed with distilled water for 5 min, and decanted. This cycle was repeated three times.

## Supplementary Materials

The following are available online at http://www.mdpi.com/2310-2861/4/1/16/s1, Figure S1: SEM images of shaped inorganic salts after alternate immersion. Figure S2: EDX line analysis of UiO68CCG-Na@CP and UiO68CCG-Na@CC. FT-IR spectra of IR15CCG-Na and IR15CCG-Na@CC. Figure S3. FT-IR spectra of IR15CCG-Na and IR15CCG-Na@CC.
