# Supplementary material for: Crystal Crosslinked Gels for the Deposition of Inorganic Salts with Polyhedral Shapes
Source: Gels. 2018 Feb 6;4(1):16. doi: 10.3390/gels4010016 (PMC6318672; doi:10.3390/gels4010016)
Supplement: Supplementary file 1 [file gels-04-00016-s001.docx]

**Supporting Information**

**Crystal Crosslinked Gels for Deposition of**

**Inorganic Salts with Polyhedral Shape**

*Yumi Mochizuki, Chihiro Oka, Takumi Ishiwata, Kenta Kokado,* and Kazuki Sada**

**Table of Contents**

**Figure S1.** SEM images of shaped inorganic salts after alternate immersion. S2

**Figure S2.** EDX line analysis of **UiO68CCG-Na@CP** and **UiO68CCG-Na@CC**. S3

**Figure S3.** FT-IR spectra of **IR15CCG-Na** and **IR15CCG-Na@CC**. S4


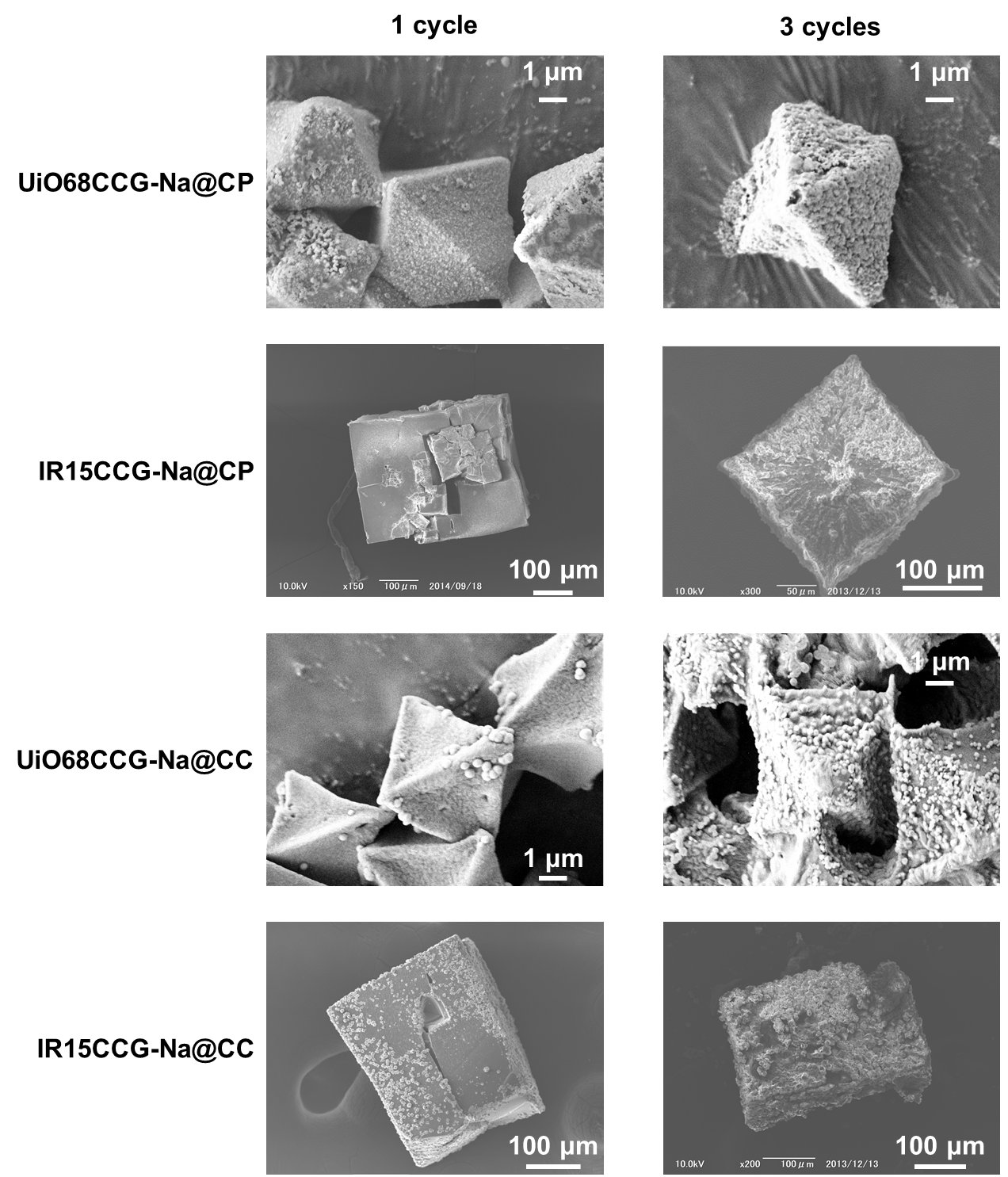


**Figure S1.** SEM images of **UiO68CCG-Na@CP**, **IR15CCG-Na@CP**, **UiO68CCG-Na@CC**, and **IR15CCG-Na@CC**, after 1 cycle and 3 cycles alternate immersion.


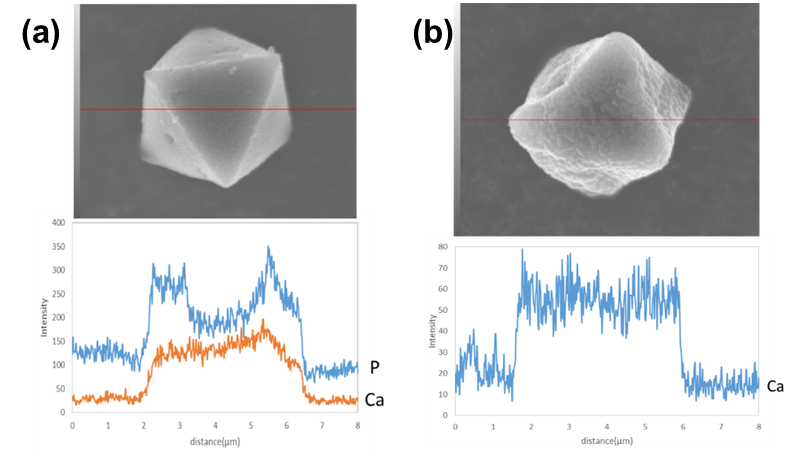


**Figure S2.** EDX line analysis of (a) **UiO68CCG-Na@CP** and (b) **UiO68CCG-Na@CC** along the thin red line.


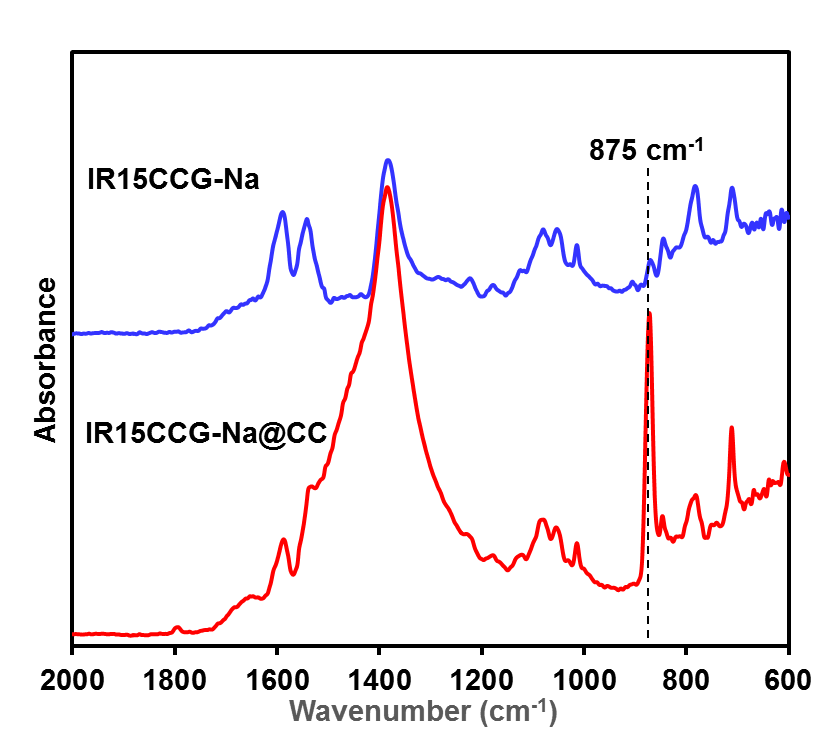


**Figure S3.** FT-IR spectra of **IR15CCG-Na** and **IR15CCG-Na@CC**.
